# Carbon monoxide effects on human ventricle action potential assessed by mathematical simulations

**DOI:** 10.3389/fphys.2013.00282

**Published:** 2013-10-17

**Authors:** Beatriz Trenor, Karen Cardona, Javier Saiz, Sridharan Rajamani, Luiz Belardinelli, Wayne R. Giles

**Affiliations:** ^1^Instituto Interuniversitario de Investigación en Bioingeniería y Tecnología Orientada al Ser Humano, Universitat Politècnica de ValènciaValencia, Spain; ^2^Cardiovascular Therapeutic Area, Gilead SciencesFoster City, CA, USA; ^3^Faculty of Kinesiology, University of CalgaryCalgary, AB, Canada

**Keywords:** carbon monoxide (CO), late sodium current (I_Na-L_), action potential (AP), early after-depolarizations (EADs), S-nitrosylation

## Abstract

Carbon monoxide (CO) that is produced in a number of different mammalian tissues is now known to have significant effects on the cardiovascular system. These include: (i) vasodilation, (ii) changes in heart rate and strength of contractions, and (iii) modulation of autonomic nervous system input to both the primary pacemaker and the working myocardium. Excessive CO in the environment is toxic and can initiate or mediate life threatening cardiac rhythm disturbances. Recent reports link these ventricular arrhythmias to an increase in the slowly inactivating, or “late” component of the Na^+^ current in the mammalian heart. The main goal of this paper is to explore the basis of this pro-arrhythmic capability of CO by incorporating changes in CO-induced ion channel activity with intracellular signaling pathways in the mammalian heart. To do this, a quite well-documented mathematical model of the action potential and intracellular calcium transient in the human ventricular myocyte has been employed. *In silico* iterations based on this model provide a useful first step in illustrating the cellular electrophysiological consequences of CO that have been reported from mammalian heart experiments. Specifically, when the Grandi et al. model of the human ventricular action potential is utilized, and after the Na^+^ and Ca^2+^ currents in a single myocyte are modified based on the experimental literature, early after-depolarization (EAD) rhythm disturbances appear, and important elements of the underlying causes of these EADs are revealed/illustrated. Our modified mathematical model of the human ventricular action potential also provides a convenient digital platform for designing future experimental work and relating these changes in cellular cardiac electrophysiology to emerging clinical and epidemiological data on CO toxicity.

## Introduction

It is now well known that carbon monoxide (CO) has significant physiological and pathophysiological effects in a number of different organ systems in mammals (Wu and Wang, 2005). This paper considers CO induced ventricular rhythm disturbances in the human heart. Recently, attention has been drawn to the fact that CO can induce cardiac arrhythmias. This is based on a number of studies in animal models and literature regarding emergency hospital admissions due to life-threatening cardiac rhythm disturbances triggered by increases in ambient levels of CO (Goldstein, 2008; Bell et al., 2009; Dallas et al., 2012). More generally, substances somewhat similar to CO, including nitric oxide (NO), and hydrogen sulfide (H_2_S) are now often referred to in the neurophysiological literature as gaseous transmitters (Leffler et al., 2006). These compounds are receiving increased attention as (i) mediators or modulators of significant, sometimes acute pathophysiological challenges, and as (ii) potential targets for therapeutic interventions (Motterlini and Otterbein, 2010).

The ability of CO to alter electrophysiological activity in the heart and nervous system has led to quite broad-ranging examinations of the ability of CO to regulate specific ion channels (Wilkinson and Kemp, 2011). These effects can occur either as direct actions of CO, or (and more commonly) CO effects on closely related intracellular signaling pathways, often involving nitric oxide signaling, metabolism, and/or downstream targets for chemical modifications of individual amino acids in functional proteins. In the cardiovascular system, specific attention has been drawn to S-nitrosylation of, specific ion channel residues (Jaffrey et al., 2001; Haldar and Stamler, 2013).

In this paper, our focus will be on the cellular electrophysiological effects of NO and related S-nitrosylation of specific sites on ion channels, ion transporters, or directly related signaling molecules (Jaffrey et al., 2001; Tamargo et al., 2010; Haldar and Stamler, 2013). This is because many cellular effects initiated by increased CO levels are mediated by downstream changes in NO and ultimately through targeted nitrosylation of defined sites (residues). At present, it is known that NO/S-nitrosylation can: (i) increase the background K^+^ current that generates the resting potential in heart (Gómez et al., 2009) (ii) activate ATP sensitive K^+^ channels in neurons (Kawano et al., 2009), and (iii) reduce a major repolarizing current (K_v_1.5) in mammalian atria (Núñez et al., 2006). In addition, a similar CO and/or NO induced reaction mechanism, as S-nitrosylation, can modulate (iv) Na^+^/K^+^ ATPase turn over in the hypoxic heart (Yakushev et al., 2012), and (v) alter release of Ca^2+^ from the sarcoplasmic reticulum (SR) by targeting the ryanodine receptor complex in heart and skeletal membranes (Gonzalez et al., 2010; Wang et al., 2010). There is also evidence that NO can increase the slowly inactivating or late Na^+^ current in nerve, muscle and heart preparations (Ahern et al., 2000; Evans and Bielefeldt, 2000).

Two recent papers (Abramochkin et al., 2011; Dallas et al., 2012) have reported CO induced changes in electrical activity and contractions, as well as induction of cardiac arrhythmias in a rat ventricle model. Evidence that a major ion channel mediated effect is an augmentation of slow inactivation of the cardiac Na^+^ current is provided. These results also emphasize an essential involvement of NO as a second messenger of this CO-induced effect. CO-induced increases in this late Na^+^ current, I_Na−L_, can significantly lengthen the action potential (AP) and result in abnormal electrical activity (early after-depolarization, EADs) characterized by repetitive firing, even after application of only one stimulus (Dallas et al., 2012). This arrhythmia can be reduced substantially following superfusion of this experimental preparation with the compound ranolazine (Dallas et al., 2012). Ranolazine, originally developed as a coronary vasodilator, is now known to be a potent and quite selective inhibitor of the I_Na-L_ in a number of different mammalian preparations (Makielski and Valdivia, 2006; Zaza et al., 2008), and also in human ventricle (Moss et al., 2008). Ranolazine effects are significant in the settings of stable angina and also in heart failure where, in both cases, it is known that I_Na-L_ is increased (c.f. Trenor et al., 2012).

The main goal of our mathematical simulations is to illustrate, using a current mathematical model of the AP in human ventricle, the consequences of a CO induced: (i) increase in I_Na-L_, (ii) NO-S-nitrosylation induced changes in I_Ca-L_, and (iii) this combination. For this purpose, CO-induced changes in I_Na-L_ that are similar to those reported in the primary experimental data (Dallas et al., 2012) were introduced into two different models of the ventricular AP and the consequences were explored systematically.

## Methods

### Human ventricular myocyte models

To simulate the electrical activity of human ventricular myocytes, two relatively recent AP models were evaluated. The Grandi et al. model (Grandi et al., 2010) is perhaps the most detailed mathematical model that is available, when judged in terms of its comprehensive ionic current portfolio and inclusion of mathematical expressions for the Ca^2+^ transient and Ca^2+^ homeostasis in the human ventricle. For the purpose of simulating some aspects of heart failure we have previously modified and utilized the original Grandi et al. model (Trenor et al., 2012). In this paper, the slowly inactivating component of I_Na_, which is denoted I_Na-L_, was reformulated based on experimental data. In the simulations presented here, we have made use of this code: specifically the Grandi model was employed after (i) modifying the Na^+^ current as in (Trenor et al., 2012), (ii) changing the L-type Ca^2+^ current, I_Ca-L_, or (iii) both.

Early in this study, some simulations were carried out using the latest human ventricular AP model published by O'Hara et al. (2011). This model is based on experimental data taken from 140 healthy human hearts; it encompasses the formulation of 18 ionic currents and carrier-mediated fluxes and a detailed formulation of steady-state and transient ion concentrations, including intracellular Ca^2+^ transients. This model reproduces many aspects of the electrophysiological behavior of human ventricular myocytes with high fidelity, and can simulate some AP alterations due to drug effects. However, it appears *not* to be able to generate/exhibit any EADs in response to the reported, CO induced, changes in I_Na_ or I_Ca_ or their combination. We have not explored the reasons for this in detail; however, it is likely that the net current at the level of the plateau of the AP is an area for further examination/modification.

### Simulation of CO effects

As reported in (Dallas et al., 2012), CO can reduce peak transient inward I_Na_ by as much as 50%, shift the inactivation curve in hyperpolarizing direction, and significantly increase the late sodium current I_Na-L_. Thus, in our simulation CO effects on the fast component of I_Na_ were modeled by introducing a 50% decrease in I_Na_ maximum conductance and a 5 mV shift in the hyperpolarizing direction of the two Na^+^ current inactivation relationships *h*_∞_ and *j*_∞_ (see Equation 1). In addition, I_Na-L_ was increased 2-fold compared to its values in the original Grandi et al. model (Trenor et al., 2012).

(1) $$h_{\infty \_\text{shifted}}=j_{\infty \_\text{shifted}}=\frac{1}{{\left(1+e^{\frac{V_{m}\;+\;71.55\;+\;5}{7.43}}\right)}^{2}}$$

*V_m_* is the membrane potential.

CO is known to elevate NO levels (Dallas et al., 2012), and this “second messenger” can have important effects on L-type Ca^2+^ current, I_Ca-L_, in the mammalian (ferret) heart, as documented in the detailed studies of Campbell et al. (1996). These investigators reported a significant increase in I_Ca-L_ (30–50%) under conditions of NO induced S-nitrosylation of this Ca^2+^ channel α-subunit. In addition, this paper also reported a nitrosylation induced change in I_Ca-L_ gating—specifically a small but significant change in the voltage dependent activation relationship—an approximately 6.5 mV shift in the hyperpolarizing direction. To reproduce these effects using the Grandi et al. model (Grandi et al., 2010), we have made a number of changes in these parameters. The most favorable/realistic results were obtained when the maximum conductance for I_Ca-L_ was increased by 20% and its activation gate was shifted 3 mV in the hyperpolarizing direction (see Equation 2). The changes applied were slightly smaller than the ones reported experimentally, but were sufficient to trigger EADs, as shown in Figure 2.

(2) $$d_{\infty \_\text{shifted}}=\frac{1}{1+e^{\frac{V_{m}\;+\;5+\;3}{6}}}$$

*V_m_* is the membrane potential.

### Simulation of ranolazine effects

The effects of ranolazine, at an assumed plasma level of 5–10 μM, were simulated by decreasing the maximum I_Na-L_ conductance by 50% (see Figure 4) (cf. Trenor et al., 2013). This maneuver was based on the fact that the recommended adult dosage level for ranolazine is in the 3–8 μM range (Belardinelli et al., 2006).

### Stimulation protocols

AP simulations were conducted at a stimulation rate of 1 Hz. Measurements were taken on stimulated output data only after achieving steady-state conditions.

### Numerical implementation

All model equations were taken from Grandi et al. (2010), and were implemented in Matlab (Mathworks Inc., Natick, MA, USA). Differential equations were solved numerically using a variable order solver (ode15s) (Shampine and Reichelt, 1997). As indicated, some simulations were performed using O'Hara et al. model, which was also implemented in Matlab. These model equations were downloaded from http://rudylab.wustl.edu, and the rapid integration methods provided in the Supplemental Materials from O'Hara et al. (2011) and one of our previous papers (Trenor et al., 2012) were used.

## Results

The initial set of computations was done for the purpose of illustrating the consequences of the CO induced changes in mammalian heart Na^+^ current, I_Na_ that were described in a primary experimental study. This paper (Dallas et al., 2012) reported that CO acting through an intracellular NO-mediated signaling cascade can (i) reduce peak I_Na_ by as much as 50% while also (ii) shifting the inactivation curve for the large transient component of I_Na_ in hyperpolarizing direction, and (iii) significantly increasing the slowly inactivating component of I_Na_ (which is denoted I_Na-L_).

**Figure 1:**
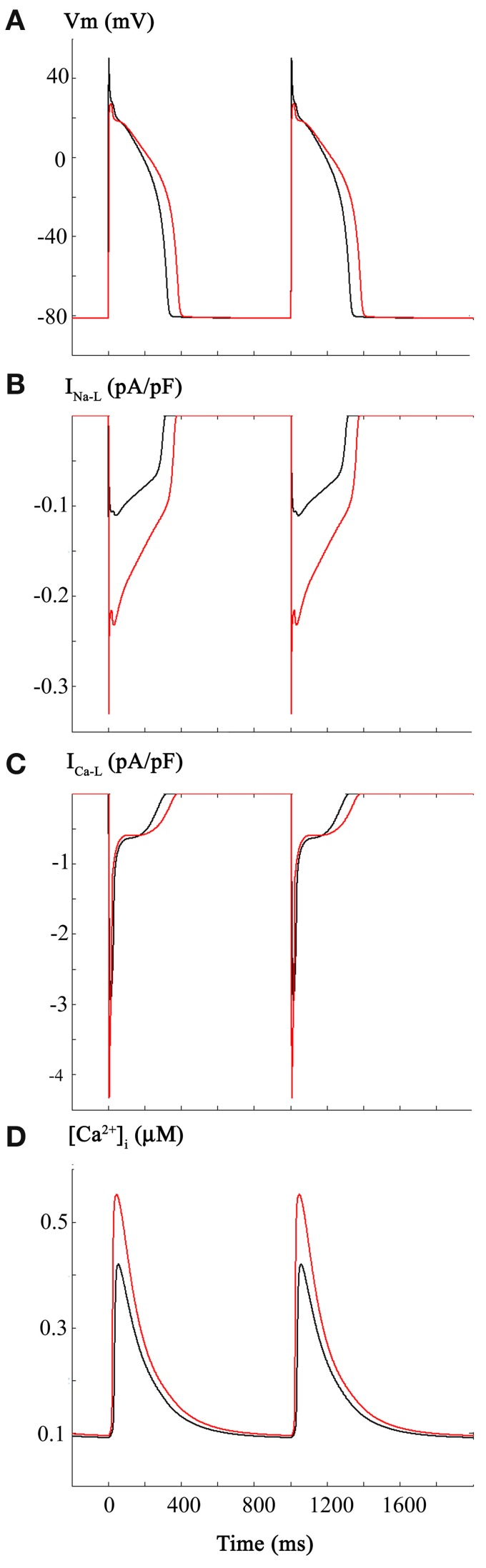
**Effects of carbon monoxide (CO) induced changes in Na^+^ current on the action potential in a human ventricular myocyte**. In this and all subsequent Figures, the Grandi et al. mathematical model of the human ventricular action potential was employed (see Methods). Control simulations are shown as black traces and simulations generated by selected changes in model parameters are shown in red. Panel **(A)** consists of control action potentials (black, 1 Hz., steady state) together with superimposed action potentials (red) that were elicited by the same stimulus parameters after: (i) reducing peak Na^+^ current by 50%, (ii) shifting the steady state inactivation relationship 5 mV in the hyperpolarizing direction, and (iii) altering the mathematical descriptors that govern the slowly inactivating or late sodium current, I_Na-L_, such that its size was increased 2-fold. As expected, these changes reduced excitability and decreased the dV/dt of the action potential upstroke as well as lengthening the AP at both APD-30 and APD-90. I_Na-L_ records are shown in Panel **(B)**. In Panels **(C,D)** the corresponding L-type Ca^2+^ current, I_Ca-L_, and intracellular calcium transient, [Ca^2+^]_i_ are illustrated. Note that these changes are indirect, i.e., they result from the changes in APD waveform and the intrinsic biophysical properties of I_Ca-L_ and the Ca^2+^-induced Ca^2+^ release (CICR) mechanisms.

The two superimposed human ventricular APs in Figure 1A illustrate the control AP waveform at 1 Hz, and the AP elicited following the changes in I_Na_ and I_Na-L_ described above. For these computations, the steady-state inactivation curve was shifted in the hyperpolarizing direction by 5 mV and I_Na-L_ was increased 2-fold. Figure 1B shows the changes in I_Na-L_; Figure 1C illustrates the indirect effects on I_Ca-L_; that is, the AP waveform lengthened, and this changed the I_Ca-L_; and Figure 1D shows the computed intracellular Ca^2+^ transient, [Ca^2+^]_i_.

In summary the reported changes in I_Na_ and I_Na-L_, when combined, result in predictable decreases in AP rate-of-rise (not shown) and small increase in AP duration. However, these changes fail to elicit spontaneous firing (EADs on DADs) or produce any correlates of arrythmogenesis. Although this pattern of results was somewhat unexpected, it is important to recall that the primary experimental data on CO were obtained in rat hearts (Ahern et al., 2000; Dallas et al., 2012), and not from human ventricular tissue or myocytes. We therefore, continued to attempt to illustrate and further understand the electrophysiological effects of CO on the human ventricular myocardium by considering additional variables.

In fact, studies of NO-mediated effects in heterologous expression systems utilizing the alpha subunit of the human heart Na^+^ channel, Na_v_1.5 (Ueda et al., 2008), reveal a somewhat different pattern of results. These include (i) an (approximately 30%) increase in peak inward Na^+^ current, a (ii) small hyperpolarizing shift in the steady-state inactivation curve for I_Na_, (iii) a marked enhancement (approximately 2-fold) in I_Na-L_ and (iv) an essential NO-induced S-nitrosylation of a defined residue on Na_v_1.5. These changes, when introduced into the Grandi model, also failed to result in increased arrhythmogenesis (results not shown).

**Figure 2:**
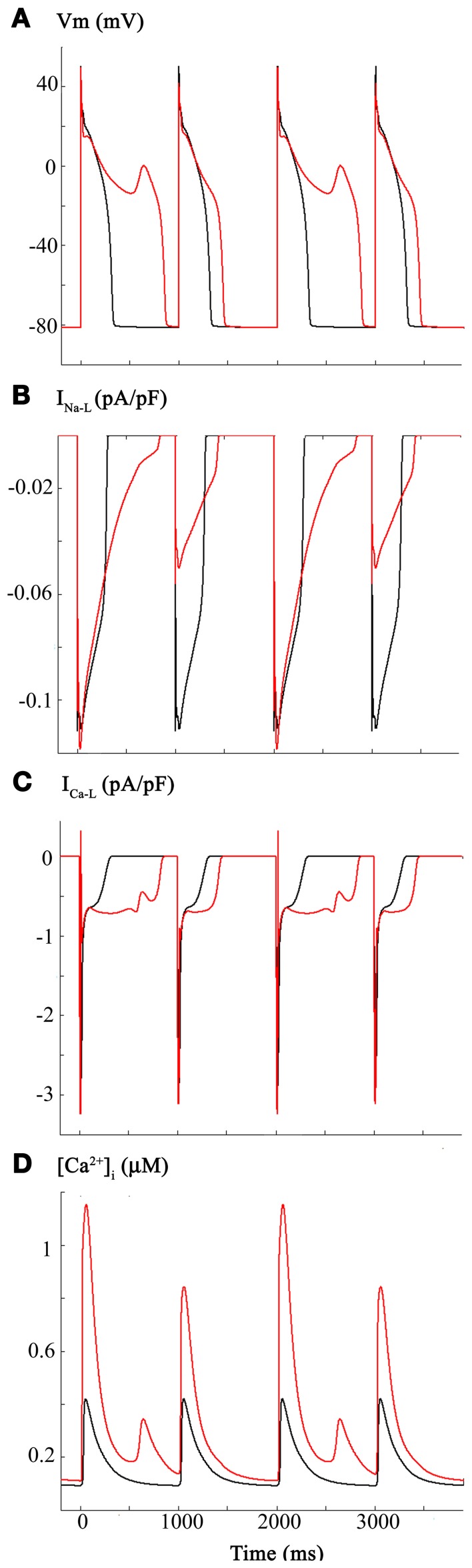
**Effects of CO-induced changes in L-type Ca^2+^ current on the human ventricular myocyte**. Based on the work of Campbell et al., I_Ca-L_ was increased 50% and the voltage dependence for the activation curve was shifted in the depolarizing direction by 3 mV. As shown in Panel **(A)** these changes resulted in a lengthening of the action potential plateau and appearance of associated oscillatory potentials or early after depolarizations (EADs). Panel **(B)** shows the changes in I_Na-L_. The longer APD results in a protracted I_Na-L_; the alterations in amplitude of this current are due to the voltage dependence of its reactivation. As shown in Panel **(C)**, these changes in the biophysical parameters that regulate I_Ca-L_ resulted in a change in peak amplitude and perhaps more importantly a significant alteration in its time course of inactivation. I_Ca-L_ also reactivates under these conditions. Panel **(D)** shows the control Ca^2+^ transient and the computed changes in [Ca^2+^]_i_ that occur as a consequence of alterations in the I_Ca-L_.

We were aware of the detailed studies of Campbell et al. (1996) on the effects of NO on I_Ca-L_, in the mammalian (ferret) heart. These investigators reported a significant increase in I_Ca-L_ (30–50%) under conditions of NO induced S-nitrosylation of this Ca^2+^ channel α-subunit. Importantly, this paper also reported a nitrosylation-induced change in I_Ca-L_ gating, specifically, a small but significant shift in the voltage dependent activation relationship—approximately 6 mV in the hyperpolarizing direction. The superimposed APs in Figure 2 illustrate the consequences of introducing these changes into the original Grandi et al. model (Grandi et al., 2010). Panel **(A)** shows the AP waveforms (black, baseline, or control) (red, following I_Ca-L_ changes). I_Na-L_ and I_Ca-L_ records are shown in Panels **(B,C)**, respectively. The L-type Ca^2+^ current is shown in Panel **(C)**. As shown in Panel **(D)**, these changes in I_Ca-L_ also resulted in a large increase in [Ca^2+^]_i_, and the [Ca^2+^]_i_ exhibited pronounced frequency dependence or alternans (see Discussion and Clark et al., 1996; Bouchard et al., 2004).

Detailed inspection of Figure 2 and comparison of this pattern of results with those in Figure 1 reveal (Panel **B**) that I_Na-L_ now shows pronounced changes in peak amplitude, even in response to the fixed stimulus rate at 1 Hz. Presumably, this arises from the markedly prolonged AP and intrinsic voltage-dependence of reactivation of I_Na-L_ (see Discussion).

**Figure 3:**
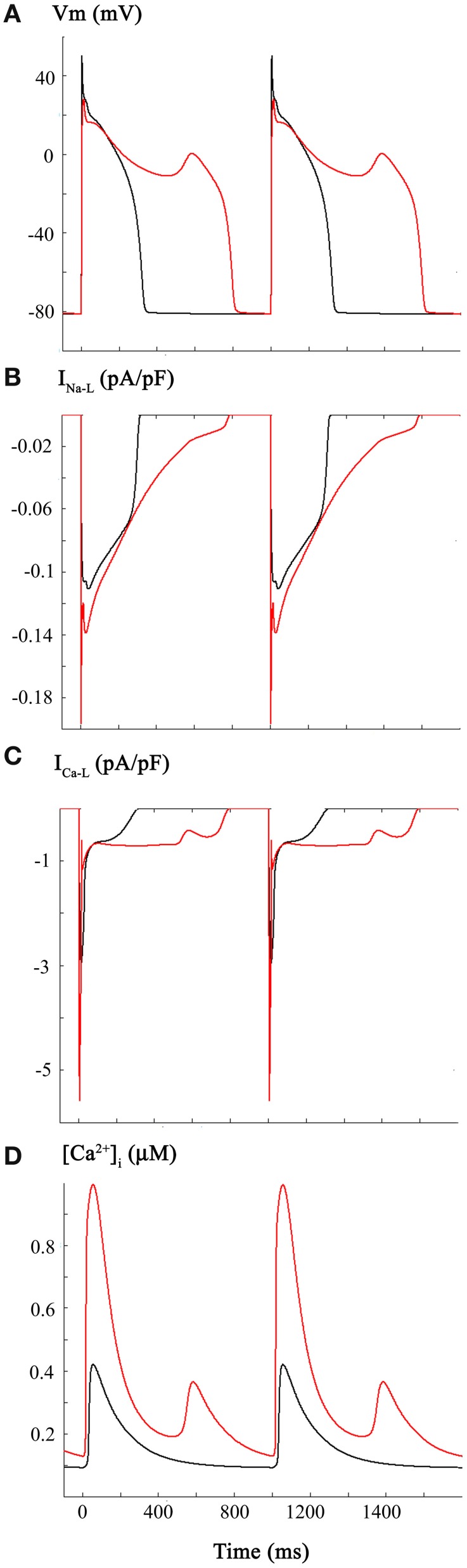
**Changes in the human ventricular action potential as a consequence of CO-induced changes in both I_Na_ and I_Ca-L_**. The layout and color-coding of this Figure is identical to that described for Figures 1, 2. As shown in Panel **(A)** when the CO-induced changes in Na^+^ and Ca^2+^ currents are combined there is marked lengthening of the action potential and a significantly increased incidence and frequency of EADs occurring during the somewhat depolarized plateau phase of the action potential. I_Na-L_ records are shown in Panel **(B)**. Panels **(C,D)** show the changes in I_Ca-L_ and [Ca^2+^]_i_. Note that the changes in I_Ca-L_ differ from those in Figure 2 as a result of the alterations in action potential waveform caused by the combined changes in I_Na_ and I_Ca-L_.

The third set of computations in this study combined the reported CO induced changes in I_Na_ and I_Na-L_ (Figure 1), with those for I_Ca-L_ (Figure 2). The resulting very significant changes in AP duration (Panel **A**), I_Na-L_ (Panel **B**), I_Ca-L_ (Panel **C**), and [Ca^2+^]_i_ (Panel **D**) are shown in Figure 3. This pattern of results was similar to, but not identical with Figure 2. In particular, the majority of the APs elicited at a physiological stimulus rate (1 Hz) exhibited marked prolongation (approximately 100%), with the appearance of an EAD late in the prolonged plateau phase. Note that under these starting conditions, the combined inward currents due to I_Na-L_ and I_Ca-L_ “held” the membrane potential of the plateau near 0 mV in contrast with the approximately −10 mV plateau level in Figure 2.

The pattern of changes shown in Figure 3 is not surprising. It is now well known that abnormal, slow depolarizations, which are denoted EADs, can be elicited by changes in I_Ca-L_ (Marban et al., 1986; January and Riddle, 1989, for review see Clusin, 2003). In fact, in rabbit ventricle a recent report from the Weiss laboratory (Madhvani et al., 2011) has illustrated in detail ways in which even very small changes in the voltage dependence of the activation of I_Ca-L_ can give rise to EADs. This is mainly, but not entirely due to the fact that there is an increased inward current at or very near the range of membrane potentials of the plateau of the normal or prolonged AP. The underlying biophysical mechanism(s) involves the non-linear interactions of the small overlapping Ca^2+^ channel—and Na^+^/Ca^2+^—exchanger mediated currents in this range of membrane potentials (Madhvani et al., 2011, for review see Fink et al., 2006). The altered AP waveform and related pro- or anti-arrhythmic effects are sometimes described in terms of an altered “repolarization reserve” at defined time-points of early or late repolarization (Wu et al., 2009; Varró and Baczkó, 2011; Trenor et al., 2013).

**Figure 4:**
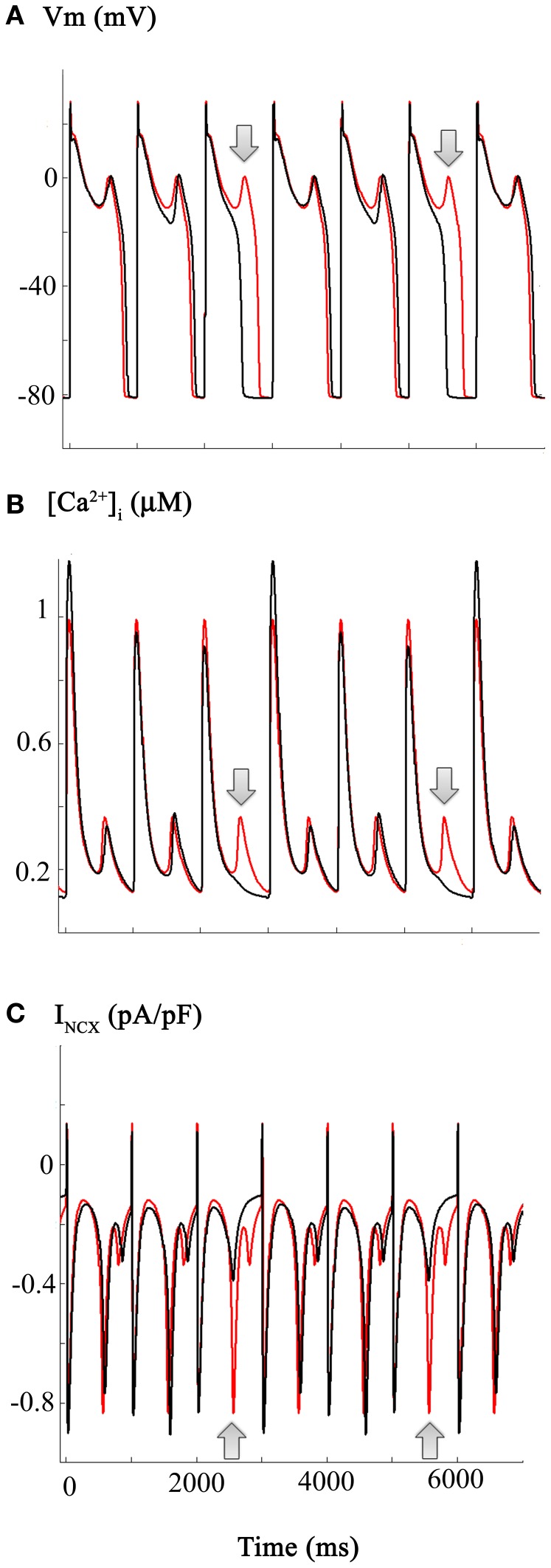
**Estimation and illustration of the effects of ranolazine on CO-induced changes in the action potential and underlying I_Na-L_, I_Ca-L_, and Na^+^/Ca^2+^ exchange currents (I_NCX_) in human ventricle**. The effects of ranolazine (shown in black) were modeled as a selective 50% reduction in the I_Na-L_. Note that this reduces action potential, duration of every 3rd action potential, as well as decreasing the incidence and the frequency of EADs. However, these changes are complex. APD (Panel **A**) as well as [Ca^2+^]_i_ and I_NCX_ records all show variations or alternans, even at this fixed stimulus rate (1 Hz). Panel **(C)** illustrates the corresponding ranolazine-induced changes in the I_NCX_. Note that in the action potential traces showing a ranolazine-induced shortening of APD, there is no spontaneous inward I_NCX_ current (see also Figure 5 and Results). The arrows denote the “extra” EADs (Panel **A**), and underlying [Ca^2+^]_i_ transient (Panel **B**), and I_NCX_ (Panel **C**) records.

If environmental or tissue-derived CO can induce life-threatening ventricular rhythm disturbances through a mechanism involving enhancement of I_Na-L_, it is plausible that, as mentioned in the Introduction, ranolazine could be effective in Critical Care settings (Dallas et al., 2012, cf. Belardinelli et al., 2006; Bell et al., 2009). The mathematical simulations shown in Figure 4 address this possibility by illustrating the effects of ranolazine “*in silico*.” These computations were carried out under starting conditions (i.e., an initial parameter set) almost identical to those for Figure 3. However, for these computations it was assumed that ranolazine actions would be the equivalent of a 50% reduction of the CO induced increase in I_Na-L_ (see Figure 1). The results show that if ranolazine (at an assumed plasma level of 5 μ M) did block approximately 50% of I_Na-L_, it could shorten the AP elicited by a 1 Hz train of stimuli. Note, however, that this partial block of I_Na-L_ reduced APD only (approximately) every 3rd AP in this 1 Hz AP train. An additional effect is also apparent: reducing I_Na-L_ changes the (i) waveform and (ii) the most negative membrane potential in some repolarizing components within this train of AP's. This primary “effect” appears to be linked functionally to the approximately normal repolarization of the next AP. In summary, when ranolazine is introduced *in silico*, the resulting AP waveforms show complex alternating patterns. However, there is an increased number of (i) near normal AP waveforms and (ii) monotonic [Ca^2+^]_i_ records. The incidence of EADs is also reduced, consistent with the previously described actions of this drug in the settings of free radical challenge, genetic abnormalities in Na^+^ channel biophysics, hypoxia, or global ischemia/heart failure (Fink et al., 2006; Trenor et al., 2012).

**Figure 5:**
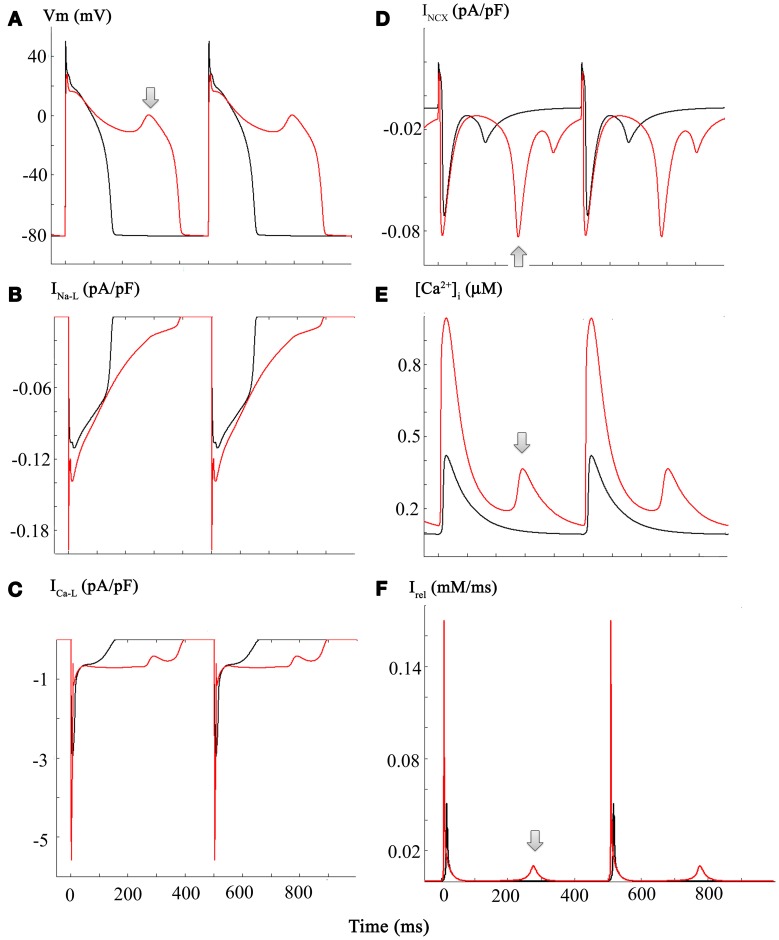
**Diagram illustrating a plausible mechanism for generation of EADs in our modified Grandi model of the human ventricular action potential**. Panels **(A** through **C)** each consist of a pair of superimposed records for the AP, I_Na-L_, and I_Ca-L_ records, respectively. Those in black correspond to control or baseline conditions, and the CO-induced changes are shown in red. Important new information is illustrated in the corresponding (i) I_NCX_ currents, Panel **(D)**; (ii) [Ca^2+^]_I_ data, Panel **(E)**; and the current generated by Ca^2+^ release from the sarcoplasmic reticulum I_rel_, Panel **(F)**. The arrows denote the EAD (Panel **A**) and underlying spontaneous Ca^2+^ release from the SR (Panel **F**), and I_NCX_ current driven by the increase in [Ca^2+^]_i_ (Panel **E**). Refer to Results and Discussion sections for further details and explanation.

The simulations in Figure 5 address and attempt to illustrate some of the underlying causative factors for EAD generation within the framework/limitations of this model of the human ventricle AP. EAD generation requires the membrane potential to be relatively depolarized, so that L-type Ca^2+^ channels can be activated and contribute a small but significant inward Ca^2+^ current. We note that the main interacting (or overlapping) ion channel mediated currents at the plateau of the AP are: (i) I_Na-L_, (ii) I_Ca-L_, (iii) I_K1_, a time-independent or background inwardly rectifying current, and (iv) the delayed rectifier K^+^ current, I_K−R_ (Trenor et al., 2013). In the presence of an enhanced I_Na-L_ the AP plateau is somewhat depolarized and the AP tends to be prolonged. In addition, under these conditions I_NCX_ generated by the Na^+^/Ca^2+^ exchange mechanism can be functionally important. A key question is: are the pronounced changes in [Ca^2+^]_i_ the cause or the consequence of the EAD/CO-induced pro-arrhythmic substrate. A plausible answer can be obtained by consideration of the results in Panels (**A–F**) of Figure 5.

Panel **(A)** shows two superimposed pairs of APs, each elicited at steady state (1 Hz) before (black) and after (red) introducing the changes shown in Figure 3. Panels **(B,C)** show the corresponding I_Na-L_ and I_Ca-L_ records. Although this information is redundant with that in Figure 3: here, it provides a means for direct comparison with the new data: Panel **(D)**, [Ca^2+^]_i_ records; Panel **(E)**, I_NCX_; and Panel **(F)**, the Ca^2+^ content of the SR.

Taken together, these results suggest that in this model the CO/NO-induced changes in I_Ca-L_ are critical. With each stimulus, a large “extra” Ca^2+^ influx prolongs the plateau and results in enhanced Ca^2+^ release and a much larger increase in [Ca^2+^]_i_. This change in [Ca^2+^]_i_ can stimulate the I_NCX_. Perhaps more importantly, however, this time-dependent and cumulative change in myocyte Ca^2+^ homeostasis results in markedly increased Ca^2+^ in the SR. Eventually, this causes anomalous Ca^2+^ release from the SR, and the Na^+^/Ca^2+^ exchanger (denoted by arrows) responds by generating an inward current that gives rise to the EAD. Under these conditions, approximately every second AP is associated with spontaneous release at the steady-state stimulus rate of 1 Hz.

This scheme/scenario seems plausible, since similar patterns of changes have been described experimentally. However, it may not be readily apparent how ranolazine, acting only on I_Na-L_ could be anti-arrhythmic (see Discussion). In brief, the answer appears to be that ranolazine, by blocking I_Na-L_, can shorten the AP. This reduction in APD results in reduced Ca^2+^ influx through I_Ca-L_ during each AP and thus, there is less Ca^2+^-loading of the SR. The end result is that SR Ca^2+^ content does *not* reach the abnormal levels required for anomalous and spontaneous Ca^2+^ release. Hence, there is no intracellular Ca^2+^ “trigger” for the electrogenic I_NCX_, that has an important role in initiating the EAD in this model (Houser, 2000; Clusin, 2003).

## Discussion

### Summary of main findings

Our mathematical modeling illustrates one set of conditions under which human ventricle myocytes can respond to a significant CO induced challenge (environmental or intrinsic) by generating an aberrant cellular electrophysiological pattern of responses, denoted EADs.

When using a modified Grandi et al. modeling framework (Grandi et al., 2010), the reported (Dallas et al., 2012) changes in Na^+^ current following CO exposure were *not* sufficient to provide the required pro-arrhythmic substrate. Additional changes in I_Na-L_ parameters may have given rise to EAD waveforms. However, we evaluated the possibility that other ion channel targets were involved in EAD initiation. Accordingly, our analyses of CO-induced EADs include changes in I_Na-L_ and I_Ca-L_. As shown in Figure 2, even very small changes in I_Ca-L_ can produce EAD generation. Although this pattern of results has not been reported when CO is used as the “toxic” stimulus; it is known that NO is a primary mediator of CO-induced pro-arrhythmic events in the heart (Dallas et al., 2012). Campbell et al. (1996) have established that NO driven S-nitrosylation can modify I_Ca-L_. We adopted this approach and were guided by this comprehensive biophysical/molecular pharmacological data set.

It is interesting that in spite of an apparent “double hit” being required for the initiation of EADs, *in silico*, reduction of only one of these contributing factors, a 50% decrease of I_Na-L_, resulted in a reduction in the EAD incidence (see Figure 4). This is consistent with the experimental observation that selective pharmacological inhibition of I_Na-L_ can serve as an antidote to CO-induced rhythm disturbances in rat ventricle (Dallas et al., 2012).

### Mechanistic insights

Some aspects of the cellular/biophysical mechanism(s) responsible for EAD generation can be deduced/confirmed by careful inspection of the new data from our simulations. EADs are slow, small depolarizations that arise in the ventricle (and in Purkinje tissue) within a transmembrane voltage range corresponding to the plateau of the ventricular AP (approximately −10 to +10 mV). A net inward current is required, and the work from the Marban (Marban et al., 1986) and January (January and Riddle, 1989) Laboratories has established that there is an obligatory requirement for activation of I_Ca-L_. In fact, I_Ca-L_, in most cases, must reactivate. That is, a small fraction of the same I_Ca-L_ channels that have opened previously in the AP to produce the distinctive plateau of the human ventricular AP open again. This produces a “pedestal” or apparently non-inactivating I_Ca-L_ component (Corrias et al., 2011). Our simulations are interesting in this regard. Detailed inspection of Figures 2–4 in fact show that EAD generation in this model occurs at a time when the I_Ca-L_ pedestal shows a small, transient *decrease*. In the Grandi et al. model, this is due to the specific mathematical expression of [Ca^2+^]_i_ dependent inactivation that is employed.

However, the non-inactivating component of I_Ca-L_ and the inward current due to I_Na-L_ markedly prolong the AP, and this change in APD waveform results in a significant increase in Ca^2+^ influx since I_Ca-L_ does not fully inactivate. This extra Ca^2+^ influx, results in a time-dependent and marked increase in SR Ca^2+^ content. Eventually this causes a spontaneous and intermittent (anomalous) pattern of SR Ca^2+^ release. Each of these increases in [Ca^2+^]_i_ gives rise to a significant “new” inward current generated by the sarcolemmal Na^+^/Ca^2+^ exchanger mechanism.

It is worth noting that during CO challenge (intoxication and recovery) or in any setting involving stress, the known effects of stimulation of the sympathetic nervous system would be expected to augment both Na^+^ and Ca^2+^ currents. These changes, alone or in combination, would enhance I_Ca-L_ and perhaps I_Na-L_, both of which would contribute a net inward current at the plateau level of the ventricular AP. The Koren Laboratory (Liu et al., 2012) has recently developed a robust model of EAD induced arrhythmias based on genetically targeted malfunction of distinct repolarizing K^+^ currents in adult rabbit ventricle. Their findings provide interesting insights into underlying mechanisms of pro-arrhythmic impulse generation at relatively depolarized (AP plateau) levels in the ventricular myocardium. Specifically, they have reported enhanced EAD activity either (i) following augmentation of I_Ca-L_ with isoproterenol, or (ii) a reduction of repolarizing K^+^ current(s) during hypokalemic conditions. Both of these maneuvers can alter the net current at the plateau of the AP and thus, change the repolarization reserve during the early repolarization phase of the AP (cf. Fink et al., 2006; Sarkar and Sobie, 2011; Trenor et al., 2012). Further insights into the ionic mechanisms responsible for EAD initiation and maintenance requires definition of, and agreement on, the range of membrane potentials at which those small oscillating events occur (Gaur et al., 2009).

### Future directions

As indicated in the Introduction, there are a number of additional targets or sites, at which CO or CO-generated signaling molecules are known to act. Perhaps the most important of these are the K^+^ currents that are expressed throughout the mammalian heart, and have important roles in setting the resting potential and modulating repolarization. The Tamargo Group (Gómez et al., 2009) has shown that the K^+^ conductance which underlies the resting potential in atrium and ventricle, K_ir_2.1 or I_K1_, is a target for S-nitrosylation. This chemical modification of this current results in increased I_K1_. Although I_K1_ is non-linear, it increases the repolarization reserve during final repolarization, and may cause a small (1–3 mV) hyperpolarization of the resting potential (cf. Fink et al., 2006). More detailed studies of CO effects should include this target of CO-mediated channel effects.

Tamargo et al. (Núñez et al., 2006) have also reported that a prominent repolarizing current in the atrium of most mammalian species, K_v_1.5, can be altered by S-nitrosylation. K_v_1.5 is decreased by S-nitrosylation, and this would be pro-arrhythmic. We note however, that K_v_1.5 is a small current in human ventricle (cf. Fink et al., 2006; Grandi et al., 2010).

As noted in the Introduction, the Makielski Group (Ueda et al., 2008) has reported that NO and an associated S-nitrosylation modification of I_Na_ can result in a significantly enhanced I_Na-L_. In fact, however, their investigation focused on NO effects on I_Na_ in the setting of an identified mutation in syntrophin that results in one variant of a pattern of ventricular rhythm disturbances, denoted the long Q-T syndrome (cf. Moss et al., 2008; Thomas et al., 2008).

It has been proposed (Dallas et al., 2012) that consideration should be given to utilization of ranolazine or ranolazine-like compounds in clinical settings (Emergency or Critical Care Medicine) that involve documented CO poisoning and involve ventricular rhythm disturbances. Improved clinical interventions directed toward minimizing cardiac risk during the required recovery period from CO intoxication are much needed (Goldstein, 2008; Motterlini and Otterbein, 2010). In principle, and as illustrated, ranolazine or ranolazine-like compounds could be considered for this purpose (Belardinelli et al., 2006; Kinobe et al., 2008; Saint, 2008; Wu et al., 2009). Ranolazine quite selectively targets I_Na-L_. However, the net current change that is the direct result of ranolazine interaction with Na^+^ channel, is integrated in the overall cardiac electrophysiological system (biophysical mechanism) for maintenance and generation of the plateau of the AP, in a way that involves complex, often non-linear interactions of a number of different currents (Fink et al., 2006). Thus, a monotonic dose-response relationship for ranolazine effects is not anticipated in this type of clinical/therapeutic setting/application and individual differences in the electrophysiological substrate are expected to/need to continue to be considered (Sarkar et al., 2012).

The computations presented in this paper have all been carried out assuming that the CO target is a single ventricular myocyte. Although this is a valid starting point, it is known that the isolated single human ventricular myocyte has electrophysiological characteristics somewhat different than the same myocyte functionally joined to other myocytes through gap junction or “placed” in a syncytium consisting of many other similar or identical myocytes. Often, the AP waveform in the syncytium is slightly but significantly different than that in a single cell. For example, the plateau “height” is less depolarized in the syncytium. Intracellular current flow, and other factors, such as intrinsic electrophysiological heterogeneity of the myocytes, can also result in the AP of a myocyte within a syncytium having a more stable resting potential together with much less intrinsic action APD variability (Huelsing et al., 2000; Zaniboni et al., 2000; Spitzer et al., 2006). In addition, it is now known that in both the ventricular and the atrial myocardium the fibroblast/myofibroblast cell population (Wu and Wang, 2005) may produce significant electrotonic effects (Maleckar et al., 2009, cf. Baudino et al., 2006). It is possible that either CO or its immediate downstream signaling molecules could alter fibroblast electrophysiology/paracrine function. One or more of these factors may alter the pattern of response which we have observed in our *in silico* investigation of CO effects.

### Limitations

We are aware that as presented our analysis of CO effects on the human ventricle is incomplete. Primary reasons for this include:

1. The computational platform that we have used is comprehensive and has been tested in terms of its ability to illustrate the functional roles of many of the individual ion channel currents that underlie the AP. However, it and most other such models have limitations. In this paper, we illustrate pronounced effects on AP waveforms and pro-arrhythmic patterns of response that arise from very small current changes that would be consistent with CO effects. The Grandi model (Grandi et al., 2010) can be relied on as a computational platform for human ventricular myocytes when exploring problems that can be addressed with semi-quantitative endpoints. However, caution is warranted when the input data suggests only e.g., a 1–3 mV shift in an activation parameter (cf. Terkildsen et al., 2008).
2. It is apparent that simulation of CO-mediated electrophysiological effects requires detailed understanding of each major component of Ca^2+^ homeostasis in the human ventricular myocyte. Recent publications show that S-nitrosylation can alter ryanodine receptor function and change Ca^2+^-induced Ca^2+^ release (Ahern et al., 2000; Wang et al., 2010; Cutler et al., 2012). Our work does not address this possibility. In fact, in the Grandi et al. modeling environment (Grandi et al., 2010) detailed studies of the consequences of changes in Ca^2+^-induced Ca^2+^ release (CICR) are not possible since many of the known subcellular details of CICR release are not included in this computational platform. After this model is developed further and e.g., the existing electrophysiological components are integrated with known properties of E-C coupling and mechanical activation (Tao et al., 2011), further insights into CO-mediated effects may be able to be obtained and Ca^2+^-pump (SERCA 2) mediated relaxation.
3. Detailed studies of CO-mediated alterations of mammalian cardiac electrophysiological phenomena are relatively recent. At present, there is incomplete information on clinically relevant plasma levels, interspecies variations, and specific intracellular signaling cascades. This information is essential for more extensive simulations that could address cellular or molecular mechanisms for CO-I_Na_-NO interactions (Ueda et al., 2008), including those arising from ion channel mutations (Thomas et al., 2008) or changes in intercellular coupling (Haas and Landisman, 2011), identification or selection of therapeutic agents, and improvement of Systems Biology (Winslow et al., 2011) approaches to human cardiac sudden death in humans.
4. Our work is not intended to detract from or replace well-established principles of CO generation or action (Maines, 1997) based on the heme oxygenase system. However, two points are noteworthy. (i) It is now known that an essential site of action of CO in resistance vessels is a reduced heme moiety that is co-localized with the Ca^2+^-activated K^+^ channels that modulate CO-induced vasodilation (Jaggar et al., 2005). (ii) CO challenge results in compromised haemoglobin function and pronounced hypoxia. Hypoxia is an established condition that results in augmented I_Na-L_ in the myocardium (Ju et al., 1996). A recent paper advances an interesting plausible scenario in which micro-anatomical integration, that is co-expression of Na^+^ channels, NO synthase isoform and G proteins are co-localized in caveolae (Besse et al., 2011).
5. Our approach to the pro-arrhythmic effects of CO challenge has placed an emphasis on EAD initiation. We acknowledge that alterations in micro- or macroscopic conduction patterns or velocities; and/or I_Na_, I_Na-L_, and I_Ca-L_ dependent changes in AP waveform have *not* been ruled out as plausible pro-arrhythmic elements. These, along with the S-nitosylation induced change in mammalian heart K^+^ currents (Gómez et al., 2009; Tamargo et al., 2010), could form the basis of a more comprehensive study, after sufficient data from one mammalian species, preferably human, is obtained.

## Author contributions

Doctors Trenor, Saiz and Giles shared responsibilities for study design. Computations were performed at Universidad Politécnica de Valencia by Doctors Cardona and Trenor, Dr. Giles wrote this manuscript. All authors contributed to final editing and the R-1 revisions.

### Conflict of interest statement

The authors declare that the research was conducted in the absence of any commercial or financial relationships that could be construed as a potential conflict of interest.
